# THRIVING OR SURVIVING? FACTORS ASSOCIATED WITH NURSING HOME SOCIAL SERVICE DIRECTORS REPORTING THRIVING AT WORK

**DOI:** 10.1093/geroni/igac059.1565

**Published:** 2022-12-20

**Authors:** Nancy Kusmaul, Mercedes Bern-Klug, Kevin Smith, Dana Cheek

**Affiliations:** University of Maryland Baltimore County, Baltimore, Maryland, United States; University of Iowa, IOWA CITY, Iowa, United States; University of Iowa, Iowa City, Iowa, United States; University of Iowa, Iowa City, Iowa, United States

## Abstract

Nursing homes (NHs) provide care to medically complex residents. Social services (SS) are primarily responsible for addressing residents’ psychosocial needs. High staff turnover in nursing homes is a significant problem. When SS staff leave, training and experience goes with them. Much research has explored nursing staff turnover, far less about SS staff turnover. Social service workers who are thriving are more focused, innovative, and engaged. This study explored characteristics contributing to NH SS directors reporting thriving at work. We surveyed 924 NH SS directors from randomly selected nursing homes. Guided by Spreitzer’s model, a hierarchical logistic regression was conducted utilizing unit contextual factors and individual agentic behaviors to examine likelihood of thriving. Unit contextual factors, i.e. being valued, feeling influence, and the NH providing quality care had the greatest influence on SS directors reporting thriving at work. These results suggest great potential for NHs to foster thriving in SS directors.

